# A Systematic Review of Research Studies Examining Telehealth Privacy and Security Practices used by Healthcare Providers

**DOI:** 10.5195/ijt.2017.6231

**Published:** 2017-11-20

**Authors:** VALERIE J. M. WATZLAF, LEMING ZHOU, DILHARI R. DEALMEIDA, LINDA M. HARTMAN

**Affiliations:** DEPARTMENT OF HEALTH INFORMATION MANAGEMENT, SCHOOL OF HEALTH AND REHABILITATION SCIENCES, UNIVERSITY OF PITTSBURGH, PITTSBURGH, PA, USA

**Keywords:** Computer security, Health personnel, Privacy, Systematic review, Telehealth

## Abstract

The objective of this systematic review was to systematically review papers in the United States that examine current practices in privacy and security when telehealth technologies are used by healthcare providers. A literature search was conducted using the Preferred Reporting Items for Systematic Reviews and Meta-Analyses Protocols (PRISMA-P). PubMed, CINAHL and INSPEC from 2003 – 2016 were searched and returned 25,404 papers (after duplications were removed). Inclusion and exclusion criteria were strictly followed to examine title, abstract, and full text for 21 published papers which reported on privacy and security practices used by healthcare providers using telehealth. Data on confidentiality, integrity, privacy, informed consent, access control, availability, retention, encryption, and authentication were all searched and retrieved from the papers examined. Papers were selected by two independent reviewers, first per inclusion/exclusion criteria and, where there was disagreement, a third reviewer was consulted. The percentage of agreement and Cohen’s kappa was 99.04% and 0.7331 respectively. The papers reviewed ranged from 2004 to 2016 and included several types of telehealth specialties. Sixty-seven percent were policy type studies, and 14 percent were survey/interview studies. There were no randomized controlled trials. Based upon the results, we conclude that it is necessary to have more studies with specific information about the use of privacy and security practices when using telehealth technologies as well as studies that examine patient and provider preferences on how data is kept private and secure during and after telehealth sessions.

## BACKGROUND AND SIGNIFICANCE

When in-person meetings and paper-based health records are used, healthcare providers have a clear idea about how to protect the privacy and security of healthcare information. Providers see each patient in a private room and the patient records are locked in a secure office setting which is only accessible to authorized personnel. When the healthcare practice is moved to the Internet, as in the case with telehealth, and all information is electronic, the situation becomes more complex. Most healthcare providers are not trained in protecting security and patient privacy in cyberspace. In cyberspace, there are many methods that can be used to break into the electronic system and gain unauthorized access to a large amount of protected health information (PHI). Therefore, the information security and patient privacy in telehealth is at a higher risk for breaches of

PHI. For instance, from 2010 to 2015 it was found that laptops (20.2%), network servers (12.1%), desktop computers (13%), and other portable electronic devices (5.6%) made up 51 percent of data sources of all healthcare data breaches that affected more than 500 individuals (Office of the National Coordinator for Health Information Technology, 2016). PHI is highly regulated in the United States. The most familiar regulation impacting healthcare facilities and providers is the Health Insurance Portability and Accountability Act (HIPAA) of 1996 (US Department of Health and Human Services, 2013). HIPAA is a federal law that provides privacy and security rules and regulations to protect PHI. The HIPAA Privacy Rule is an administrative regulation created by the Department of Health and Human Services (DHHS). It was developed after the US Congress passed HIPAA, and went into effect in 2003.

The HIPAA Privacy Rule only applies to healthcare providers that conduct electronic billing transactions but is effective for both paper and electronic health information. It is a set of national standards that addresses the use and disclosure of PHI by a covered entity such as a healthcare organization as well as establishing privacy rights for individuals on how their PHI is used and shared. Its major objective is to protect the flow of health information while at the same time providing high quality healthcare.

The HIPAA Security Rule went into effect in 2005 and regulates only electronic health information. It is a set of national standards that protects an individual’s electronic health information that is created, received, used or maintained by a covered entity such as a healthcare organization. It requires the administrative, physical, and technical standards to be adopted so that confidentiality and integrity of electronic PHI is protected.

In addition to HIPAA, there are many other federal and state laws that govern the use and disclosure of health information. Of these laws, HIPAA and the Health Information Technology for Economic and Clinical Health (HITECH) Act of 2009 have provided the most specific regulations for the protection of privacy and security of health information in the United States. However, some state regulations may be even more stringent, such as requiring a consent form for disclosure of a patient’s own medical record when HIPAA does not require consent (Rinehart-Thompson, 2013). The HITECH Act includes changes to the HIPAA Privacy and Security rules that focus mainly on health information technology and strengthens standards for the privacy and security of health information. It went into effect in 2010 but some parts of the act have different compliance deadlines (Rinehart-Thompson, 2013).

For this article, we adopted the Health Resources and Services Administration’s (HRSA) 2015 definition of telehealth: “the use of electronic information and telecommunications technologies to support long-distance clinical health care, patient and professional health-related education, public health and health administration. Technologies include videoconferencing, the Internet, store-and-forward imaging, streaming media, and terrestrial and wireless communications” (Health Resources and Services Administration, 2015). The HRSA definition was used because it aligns with our purpose, which is to provide a systematic review of published papers that pertain to privacy and security provisions used by healthcare providers when deploying telehealth technologies in the United States.

Our previous experiences in interacting with telehealth providers suggest that the providers do not always know the best practices to use to decrease the risk of privacy and security issues in telehealth (Cohn & Watzlaf, 2012; Watzlaf, 2010; Watzlaf, Moeini, & Matusow, 2011). Many of the features within the free, consumer-based video and voice communication systems that were evaluated did not demonstrate to the providers using them that the information was private and secure (Watzlaf & Ondich, 2012). Also, many of the telehealth providers did not know the best practices to use to educate consumers on privacy and security (Watzlaf, Moeini, & Firouzan, 2010; Watzlaf, Moeini, Matusow, & Firouzan, 2011).

Through our past work, audit checklists were developed to determine if a system supports HIPAA compliance (Watzlaf et al., 2010; Peterson & Watzlaf, 2014). The 58-question checklist is specific to Information and Communication Technologies (ICTs) (Watzlaf et al., 2010). There are already methods and tools available for healthcare providers to evaluate the security and privacy features of telehealth systems they are currently using. Now, it is necessary to conduct a systematic review on the status of privacy and security provisions that are used by healthcare professionals when deploying telehealth services to see if they are using the tools and guidelines available to them or if they incorporate new systems to evaluate privacy and security within telehealth systems.

### OBJECTIVES

1. Evaluate, from published papers, what privacy and security measures were addressed when healthcare providers used telehealth technologies.
2. Compile best practices and guidelines for healthcare professionals using telehealth technologies.

## MATERIAL AND METHODS

### SEARCH STRATEGY

A systematic literature search was performed on papers published between 2003 to 2016. The sources used in the search included PubMed (Medline via PubMed; National Library of Medicine, Bethesda, MD; started in 1966) CINAHL databases (indexing from nursing and allied health literature) and INSPEC (a scientific and technical database developed by the Institution of Engineering and Technology).

Briefly, our literature search strategy combined synonyms for telehealth with privacy and security across healthcare professionals. The list of synonymous terms was voluminous. Some examples of synonymous terms for telehealth included telemedicine, telepathology, telerehabilitation; synonymous terms for privacy and security included confidentiality, encryption, access control, authentication; synonymous terms for healthcare professionals included physicians, clinicians, nurses, occupation therapists. Language restrictions included those papers written in English only. In addition, reference lists were reviewed manually from relevant original research and review papers.

**Figure 1:**
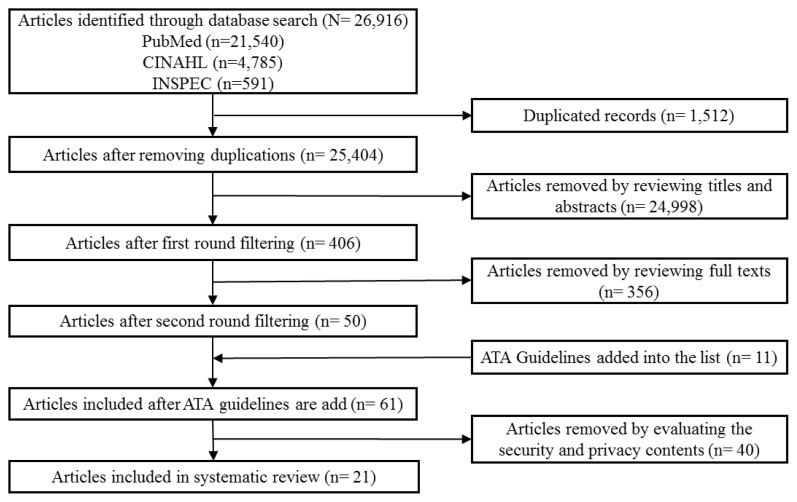
A flow diagram of the search and selection process. Figure 1 description: Figure 1 depicts a flow diagram of the search and selection process. First box a top: Articles identified through database search (N=26,916), PubMed (n=21,540), CINAHL (n=4,785), INSPEC (n-591). Arrow to box below: Articles after removing duplications (n=25,404); arrow to the box to the right: Duplicate records (n=1,512). Next arrow to box below: Articles after first round filtering (n=406); arrow to the box to the right: Articles removed by reviewing titles and abstracts (n=24,998). Next arrow to box below: Articles after second round filtering (n=50); arrow to the box to the right: Articles removed by reviewing full texts (n=356). Next arrow to box below: Articles included after ATA guidelines are added (n=61); arrow to the box to the right: Articles removed by evaluating the security and privacy contents (n=40). Last arrow to the box below: Articles included in systematic review (n=21).

These searches returned 21,540 papers from PubMed and 4,785 papers from CINAHL, and 591 papers from INSPEC for a total of 26,916 papers, of which 1,512 were duplicates. After a review of titles and abstracts, 21 papers were reviewed in full text (Figure 1). After the first round of article selections, one third of the papers were found to be international. Papers were then restricted to those in the United States since HIPAA and HITECH are laws that are enforced in the United States only and these laws are a major influence in privacy and security in the US.

The protocol for this study was based on the Preferred Reporting Items for Systematic Review and Meta-Analysis Protocols (PRISMA-P). The PRISMA-P contains 17 items that are considered essential as well as minimum components to include in systematic reviews or meta-analyses. PRISMA-P recommends that each systematic review include detailed criteria using the PICOS (participants, interventions, comparisons, outcome(s) and study design) reporting system (Moher et al., 2015). Details of the full protocol have been previously published in Prospero and the International Journal of Telerehabilitation (Watzlaf, DeAlmeida, Zhou, & Hartman, 2015; Watzlaf, DeAlmeida, Molinero, Zhou, & Hartman, 2015).

### STUDY ELIGIBILITY

To be eligible for this systematic review, published papers had to meet all the following criteria:

1. Published papers that included research, best practices, or recommendations on the use of telehealth and privacy or security.
2. Published papers that included any type of health care professional using any available telehealth for their clients with a focus on privacy and/or security, HIPAA and/or HITECH.
3. Published papers with full text in English.
4. Published papers where research or recommendations focused on the US only published between 2003–2016.
5. Existing solutions/best practices to privacy and security challenges, HIPAA compliance (qualitative and quantitative) in telehealth use.

### EXCLUSION OF PAPERS

Papers were reviewed and excluded in different phases:

- Phase I: Duplicates Removed. A total of 26,916 papers were found in the three databases and 1,512 were removed as duplicates to yield 25,404 papers.
- Phase II: Articles Removed by Reviewing Title and Abstract. A title/abstract review was conducted, first by two independent reviewers. A third reviewer was used to resolve disagreement (24,998 excluded, to yield a total of 406 papers).
- Phase III: Articles Removed After Reviewing Full Text. A full text review of 406 papers was conducted by all three reviewers (356 excluded, 50 papers remained).
- Phase IV: American Telemedicine Association Guidelines Added. Since the American Telemedicine Association (ATA) guidelines were not returned from the original search because they were guidelines and not peer-reviewed articles, they were added into the original list (50) because of their focus on telehealth, privacy and security (11 added. Total of 61 papers).
- Phase V: Articles Removed by Evaluating Security and Privacy Content. A review of these papers to examine security and privacy contents yielded 40 exclusions. And eventually, a total of 21 papers were included in the final systematic review.

In the initial title/abstract review the major reasons for exclusion were:

1. Papers were published before HIPAA was enforced in 2003
2. Studies were not conducted in the US and therefore did not abide by HIPAA/HITECH

In the full text review the major reasons for exclusion were that the papers did not include both telehealth and a major aspect of privacy and security related to telehealth use.

### DATA EXTRACTION PROCESS AND QUALITY ASSESSMENT

All search results were exported into EndNote libraries. EndNote is a bibliographic management system. De-duplications were performed by using the method described by Bramer et al (Bramer, Giustini, de Jonge, Holland, & Bekhuis, 2016). Studies were removed if they were found to be duplicated. The PDFs of the papers reviewed were stored in a shared Box account (i.e., a secure cloud content platform in which users can share large documents as well as collaborate, Redwood City, CA).

Each article meeting the inclusion criteria was reviewed and its characteristics documented using a standardized pre-tested data extraction form. The data extraction form captured the following data items: the three large goals of privacy and security (confidentiality, integrity, and availability); the specific techniques for achieving these goals (authentication, encryption, access control, physical security, policy, database backup, error detection, anti-virus, software patches, secure system design, intrusion detection); and the methods in each system (study designs, settings, and outcomes).

The reference librarian performed the search and only provided the title, abstract and year to the reviewers. The two reviewers (DD, VW) independently read the title and abstracts of the identified papers and determined eligibility based on the specified inclusion/exclusion criteria. To better know how to appropriately search the article titles and abstract, two of the reviewers (DD and VW) conducted a pilot study by using a small sample (n=100) of papers, made the selection and then discussed the results against the selection criteria. From this pilot study we could determine that we applied the same selection criteria for our search strategy.

Reviewers were blind to journals, study authors and institutions. Any disagreements between the reviewers were resolved by a third reviewer (LZ). Inter-rater reliability was measured using the Cohen’s kappa statistical test (k). An inter-rater Kappa score was assessed during the first round of the paper selection, to ensure a Kappa score at or above 0.8 as measured by Cohen’s Kappa (k) statistical test. Full-text of studies making this first cut were reviewed.

Three reviewers screened these for inclusion/exclusion criteria. Selection disagreements were resolved through discussion and reasons for excluding studies were recorded. A form, developed in Excel, was used to extract data from selected studies and included the author, year of publication, reference; study design and sample size; setting; privacy and security descriptions; primary outcomes; study limitations, HIPAA compliance, and best practices. Reviewers assessed the overall quality of evidence for every important outcome using the GRADE four point ranked scale: (4) High; (3) Moderate; (2) Low; (1) Very low (Balshem et al., 2011). Full papers were used as evidence for decisions about the quality of evidence and the strength of recommendations. Any differences in the grading were assessed and discussed in several meetings with investigators until full consensus was reached.

### DATA SYNTHESIS

Quantitative analysis of the data from the papers was limited due to the lack of quantifiable data in the privacy and security literature. However, subcategories with similar characteristics received more in-depth comparisons. Investigators first broke the data into qualitative themes that related to privacy, security and administrative content. Each of those areas were broken down into subthemes such as patient rights, use, and disclosure for privacy; technical and physical for security; and organizational and education/training/personnel for administrative. Then, specific content within the 21 papers were reviewed closely and categorized across each of those themes and subthemes.

## RESULTS

### REVIEWER AGREEMENT

For the 25,404 entries reviewed by 2 reviewers the percentage of agreement was very good with the observed value of 99.04% and the 95% CI between 98.91 to 99.16 calculated per the Wilson efficient-score method. For the Cohen’s kappa, the observed kappa is 0.7331 and the 95% CI are 0.7009 to 0.7653. Although the kappa is lower than 0.8, this still suggests substantial agreement (Fleiss, Cohen, & Everitt, 1969).

### TIME PERIOD AND TYPE OF STUDIES

A total of 21 papers (Watzlaf & Ondich, 2012; Watzlaf et al., 2010; Watzlaf, Moeini, Matusow, et al., 2011; Peterson & Watzlaf, 2014; Paing et al., 2009; Cason, Behl, & Ringwalt, 2012; Daniel, Sulmasy, & for the Health and Public Policy Committee of the American College of Physicians, 2015; Naam & Sanbar, 2015; American Telemedicine Association, 2009, 2011, 2014a, 2014b, 2016; Hall & McGraw, 2014; Garg & Brewer, 2011; Brous, 2016; Mullen-Fortino et al., 2012; Nieves, Candelario, Short, & Briscoe, 2009; Putrino, 2014; Demiris, 2004; Demiris, Edison, & Schopp, 2004) were selected for this systematic review. These selected papers were published between 2004 to 2016, in which 29 percent of them were published between 2011–2012. The papers included several telehealth specialties such as telerehabilitation, telepsychiatry, teletrauma, telenursing and tele-diabetes. Sixty-seven percent were guideline/policy/strategy type studies, with three using a survey or interview method (14%). Other studies included a usability study, a systematic review, a pilot study and an opinion piece. There were no randomized controlled trials found that focused on privacy and security in telehealth (Table 1).

### DESCRIPTIVE ANALYSIS OF ALL STUDIES

A quantitative analysis of the privacy, security, and administrative areas that were discussed in the papers is summarized in Table 2. All studies discussed some aspect of privacy and security. Sixty-seven percent addressed patient rights to include informed consent, accessibility, confidential communications, or the patient’s ability to amend their information. Thirty-eight percent addressed use and disclosure to include how video sessions are retained, authorizations for release of information to other countries, websites, and third parties, accounting of disclosures, purging and/or deletion schedule of files on mobile devices and audio and video muting to maintain privacy. Sixty-seven percent of the studies addressed the technical aspects of security to include encryption, two-factor authentication, data backup, storage and recovery to meet HIPAA requirements, National Institute of Standards and Technology (NIST) and Health Level-7 (HL7) recommendations. However, only 38 percent addressed the physical aspects of the telehealth session to include a secure server location, back-up generator and maintaining a secure physical environment for where the telehealth session is held. One of the studies contained a systematic review of telemedicine security and found poor reporting of methodologies for telemedicine technologies and security measures. Fifty-two percent of the papers did not discuss the organization of privacy and security through policies, procedures, Business Associate Agreements (BAAs) or compliance audits, however, 67 percent addressed the need for education and training of providers, patients and technical support workforce.

Table 3 provides a detailed summary of all papers for privacy, security and administrative content. Most of the patient rights content dealt with providing verbal or written informed consent in simple, easy to understand language and to have providers discuss the risks of privacy and security when using telehealth. Use of audio/video muting and a secure physical environment was also discussed to be included in the consent for treatment so that the patient understands how their information during and after the telehealth session is private. Use and disclosure was not as clearly addressed, although several papers stated that access to patient information should only be granted with proper authorization, and there was a need to have this discussed with the patient so that they understood ownership of the data before the telehealth session begins. Encryption and two-factor authentication were other major areas addressed in the papers. Some papers did provide details as to the types of encryption to use as well as meeting HIPAA and NIST requirements and recommendations. Data backups, storage of the video files, and the ability to keep them secure was also discussed. Other areas addressed included a review of consumer-based free systems and the importance of healthcare providers’ understanding of which telehealth technologies meet federal, state, and local laws. Other areas mentioned included performing an overall privacy and security assessment of the telehealth system and to maintain security solutions specific to the telehealth system, making sure that confidentiality and security are a primary concern. Many of the papers expressed the need for overall provider and patient awareness, education and training and policies on keeping telehealth information private and secure, and policies that specify who can be included in the telehealth session. Other papers expressed the need for maintaining a BAA with the vendor providing the telehealth system. Some papers addressed the need for more research on the effectiveness of telemedicine to include telehealth security training, legal liability, HIPAA compliance and the importance of an independent assessment of overall privacy and security. Some of the papers described the lack of current scientific studies around privacy and security in telehealth and the need for more studies that demonstrate the effectiveness of best practices in privacy and security of telehealth (Table 3).

## DISCUSSION

This systematic review of privacy and security practices that healthcare providers may use with telehealth technologies has shown that privacy and security is a concern across all types of specialties such as telerehabilitation, telenursing, teletrauma, and telepsychiatry. All providers need to make privacy and security of utmost concern when conducting a telehealth session.

The papers suggest that most of the work has been policy and strategy pieces with no experimental or quasi-experimental studies represented. In both survey research studies conducted, healthcare providers had concerns over privacy and security in telehealth and that it can be intrusive for the patient. In an interview study, it was found that providers did not believe that telehealth increased the risk of privacy or security concerns although some did not know enough to answer the question fully and thought there could be increased risk. These studies alone show that there is uncertainty on this topic.

Many healthcare providers may not know all the many aspects of privacy and security within telehealth and need more education and training as well as technical support personnel to help them in these areas. Many of the policy studies stated that policy and procedure (P&P) as well as education and training are needed for all healthcare providers and technical support personnel to prevent breaches of PHI.

These papers also stated that healthcare professionals need to know state, regional, and national laws and regulations, legal liability, HIPAA/HITECH and HL7 compliance, as well as measures used to ensure availability of PHI to the proper users. Methods were also discussed regarding how audio or video recordings are to be stored, maintained and accessed to protect patient privacy, and how mobile devices used in telehealth sessions are to be reinforced to protect the privacy and security of patient information.

The most detailed information surrounding informed consent for a telehealth session was found in the ATA guidelines and recommendations and discussed how maintaining privacy and security within the telehealth session must be included in the informed consent in easy to understand language especially when discussing encryption, authentication and other methods to maintain confidential communications between provider and patient. The use of audio and video muting as well as the ability to quickly change from public to private audio mode so that unauthorized users may not see or hear what is being communicated was also discussed throughout the ATA guideline papers.

There does not seem to be consensus about the use and disclosure of PHI in telehealth since some systems will allow sharing with certain groups as part of their privacy policy. HIPAA, however, states that proper authorizations are warranted when there are requests for information and an accounting of disclosures is necessary when PHI is shared. However, there still seems to be some uncertainty as to what parts of the telehealth session will be kept, for how long, how they will be maintained and where they will be stored.

If the telehealth sessions are recorded and kept with the electronic health record (EHR) then proper authorizations are necessary when PHI is requested. However, there is no standard method for how this is done. Some systems may convene a telehealth session and not store any of the information that was transmitted. Some may record the session but then destroy the recording after the session is over. Some may record and store the session or even transmit the session to a third party for additional treatment and consultation. Some type of standard process in this area is needed.

Security measures such as encryption and authentication were addressed, but not all papers provided a standardized description of the encryption methods used or the best methods for authentication. Very few papers addressed the importance of an independent audit on the telehealth system for privacy and security features by an outside entity.

## LIMITATIONS

There were some limitations to our systematic review. Due to time constraints the grey literature, such as dissertations and other unpublished reports, other databases listed in the protocol, vendors or authors (also mentioned in the protocol) were not searched. Also, as mentioned previously, only English language articles were reviewed.

## CONCLUSION

In summary, more scientific research studies are needed to determine the best practices in privacy and security surrounding telehealth. Experimental studies that address the effectiveness of privacy and security evaluations of the telehealth system, proper informed consent that discusses the privacy and security aspects of the telehealth session with the patient as well as testing of access control, disaster recovery and risk analysis of the telehealth system are essential to improve the practices of the entire telehealth team.

Best practices that are consistent across all types of telehealth services for all healthcare providers are needed to address all privacy and security issues. Privacy and security aspects are just as important as providing a clear and trouble-free telehealth session and a privacy and security evaluation should be performed before the telehealth system is used with a patient. Tools used to assist healthcare providers on what they should look for when deciding on a telehealth system are needed. This systematic review results informed the need for and subsequently led to the development of a best practice tool that will enable healthcare providers to assess privacy and security features of the telehealth technologies they are planning to use. Hopefully, this tool will move healthcare providers one step closer to enabling best practices in privacy and security in telehealth.

## Figures and Tables

**Table 1:** Overview of Reviewed Studies

Overview of Studies		
Time Period	#	%
2004–2005	2	9.5
2009–2010	4	19.0
2011–2012	6	28.6
2013–2014	5	23.8
2015–2016	4	19.0
Total	21	100
Specialties	#	%
Telepsychiatry	2	9.5
Teletrauma	2	9.5
Telenursing	2	9.5
Telerehabilitation	5	23.8
Telepathology	1	4.8
Teleburn	1	4.8
Telediabetes	2	9.5
Telesurgery	1	4.8
General telehealth	5	23.8
Total	21	100
Type of Study	#	%
Guideline/policy/strategy	14	66.7
Survey/Interview	3	14.3
Usability	1	4.8
Pilot	1	4.8
Opinion	1	4.8
Systematic/literature review	1	4.8
Total	21	100

**Table 2:** Privacy, Security, and Administrative Content

Privacy, Security, Administrative Content	#	%
**Privacy**		
*Patient Rights*		
Yes, addressed	14	66.7
No, not addressed	7	33.3
*Use and Disclosure*		
Yes, addressed	8	38.1
No, not addressed	13	61.9
**Security**		
*Technical*		
Yes, addressed	14	66.7
No, not addressed	7	33.3
*Physical*		
Yes, addressed	8	38.1
No, not addressed	13	61.9
Privacy, Security, Administrative Content	#	%
**Administrative**		
*Organization (policies)*		
Yes, addressed	10	47.6
No, not addressed	11	52.4
*Education/Training/Personnel*		
Yes, addressed	14	66.7
No, not addressed	7	33.3

**Table 3:** Detailed Summary of All Papers for Privacy, Security and Administrative Content

	Privacy		Security		Administrative	
Article Title, Year *Journal* Type of Study	Patient Rights (access, amend, right to confidential communications, informed consent etc.)	Use & Disclosure (authorizations, accounting of disclosures, de-identification of data etc.)	Technical (encryption, access control, authentication, data backup, storage, recovery)	Physical (secure server location, backup generator etc.)	Organizational (policies, BAAs, auditing)	Education/Training/Personnel
(American Telemedicine Association, 2009) *Evidence Based Practice for Telemental Health* Policy	Keep physical surroundings private using audio/video muting; Considered essential to easily change from public to private audio mode.					
(American Telemedicine Association, 2014a) *Clinical Guidelines for Telepathology* Policy	Unauthorized persons should not have access to sensitive information. Use audio/video muting, and easily change from public to private audio mode.	Consideration should be given to periodic purging or deletion of telepathology files from mobile devices.	Data transmission must be secure through encryption that meets recognized standards. Mobile device use requires a passphrase or other equivalent security feature, multi-factor authentication, inactivity timeout function with passphrase or re-authentication to access the device after timeout is exceeded (15 minutes).	Give providers the capability to use remote wiping if device lost or stolen. Back up or store on secure data storage locations. Do not use cloud services if they cannot comply in keeping PHI confidential.	Mobile devices should be kept in the provider’s possession when traveling or in an uncontrolled environment.	Those in charge of technology should know technology security. Mobile devices should be kept in the provider’s possession when traveling or in an uncontrolled environment.
(American Telemedicine Association, 2016) *Practice Guidelines for Teleburn* Policy	Informed consent: discuss with patient about the telehealth session and use simple language especially when describing all privacy and security issues such as encryption, store-forward transmissions of data/images, videoconferencing etc. Key topics should include confidentiality and limits to confidentiality in electronic communications; how patient information will be documented and stored.		If transmission data are stored on hard drive, use Federal Information Processing Standard (FIPS) 140–2 encryption AES as acceptable levels of security. Pre-boot authentication should also be used.			Those in charge of the technology should educate users with respect to all privacy and security options. Educate patients on the potential for inadvertently storing data and PHI; intention to record services; methods of storage; how PHI will be shared with authorized users and encrypted for maximum security; recordings will be streamed to protect accidental or unauthorized file sharing or transfer.
(American Telemedicine Association, 2014b) *Core Operational Guidelines for Telehealth Services Involving Provider-Patient Interactions* Policy	Healthcare providers should provide to the patient verbal/written information related to privacy and security; potential risks and confidentiality in easy to understand language, especially when discussing encryption or potential for technical failures; limits to confidential communication, documentation and storage of patient information. Should also discuss a policy for the patient sharing portions of this information with public and written agreements may be needed to protect both the patient and provider.	Access to recordings only granted to authorized users. Stream to protect from accidental or unauthorized file sharing/transfer. Privacy Features should include: audio, video muting, easily change from public to private mode, privacy of the mobile device.	Multi-factor authentication; inactivity timeout function; keep mobile devices with provider always; wipe or disable mobile device if lost or stolen. Audio, video and all other data transmission should use encryption (at least on the side of the healthcare professional) that meets recognized standards. Use software that has appropriate verification, confidentiality, and security measures. If services are recorded, store in a secure location and make accessible to authorized users only.	All devices should have up to date security software, device management software to provide consistent oversight of applications, device and data configuration and security, backup plan for communication between sites and discussed with the patient. Only allow one session to be open at one time and if there is an attempt to open an additional session the system will automatically log off the first session or block the second session from being opened. Session logs should be secured in a separate location and only granted to authorized users. Back up to or store on secure data storage locations. Do not use cloud services if they cannot comply in keeping PHI confidential.	Establish guidelines for periodic purging or deletion.	Providers should give guidance to patients about inadvertently storing PHI and how best to protect privacy. Discuss recording of services, how information will be stored and how privacy will be protected.
Brous, 2016) *American Journal of Nursing* Policy	Nurses must meet medical information and patient privacy requirements of HIPAA, as well as state privacy laws, organizational policies, and ethical standards.		Devices that contain PHI must meet security requirements, and wireless communications must have cybersecurity protection; electronic files must be stored in a manner that ensures privacy and confidentiality since audio and video recordings are susceptible to hacking.			All providers should be educated on how to prevent data breaches when communicating information via telehealth, transmitting images or audio or video files electronically and on how to respond when they do occur. To meet HIPAA standards any systems that transmit or store electronic information about patients must be operated and monitored by computer technicians with expertise in security measures. Also, all health care providers should check state privacy laws which can be more stringent than HIPAA. The National Telehealth Policy Resource Center provides state-specific information on laws, regulations, reimbursement policies and pending legislation.
(Cason et al., 2012) *International Journal of Telerehabilitation* Survey	44% of providers surveyed expressed concerns with privacy issues		40% of providers surveyed expressed concerns with security issues	Need for secure and private delivery platforms		Personnel shortages in telehealth delivery
(Daniel et al., 2015) *Annals of Internal Medicine* Policy Position Paper					Skype not considered HIPAA compliant since no BAA with Microsoft. Skype was noncompliant with Oklahoma Health Care Authority’s policy.	
(Demiris et al., 2004) *Telemedicine Journal and e-Health* Interview Research Study	18.7% need for digital images to be captured and store in EHR at point of care.					56.2% did not believe that security and privacy risks would be increased when using telemedicine; 31.2% said they did not know enough to respond to the question; and 12.5 % believed there could be increased risk. 9.3% said technical support not readily available; All providers were not influenced in their ability to ask questions due to concerns over security or privacy. Further education is needed on this topic.
(Demiris, 2004) *International Journal of Electronic Healthcare* Policy Overview	Privacy, storage, transmission of images and maintenance of video/audio recordings and other PHI must be examined and addressed as transmission of this data over communication lines have concerns of privacy violations.	Access to ownership of data must be addressed with the patient since some patients share this data with a web server owned by a third party that allows providers to log in and access their patient’s data.				
(Garg & Brewer, 2011) *Journal of Diabetes Science and Technology* Systematic Review			Many telemedicine researchers are unfamiliar with the field of security in general. The authors found instances of poor encryption standards, designs of communication protocols with no proof of security, HIPAA or HL7 compliance. Reliability and availability of the systems are key since many provide critical life supporting systems for people with diabetes and other chronic illnesses. Data integrity, the quality of security research, network security and cryptography all need to be improved as well, per this systematic review of security of telemedicine systems.			Most of the papers in the systematic review of security in telehealth did not address training, legal liability, or HIPAA and HL7 compliance. Another area that was neglected was research on availability or the measures used to ensure availability of telehealth systems.
(Hall & McGraw, 2014) *Health Affairs* Policy					No federal agency has authority to enact P&S requirements to cover the entire telehealth ecosystem and these authors advocate for the Federal Trade Commission (FTC) to do this.	
(Mullen-Fortino et al., 2012) *American Journal of Critical Care* Survey Research						179 nurses that use telemedicine were surveyed and 11% of nurses surveyed believe it is intrusive; 27% believe it decreases patient privacy and 13% believe it creates a feeling of being spied upon. It is important to change these perceptions of nurses for telehealth technology to expand.
(Naam & Sanbar, 2015) *Journal of Hand Surgery* Policy	Verbal or written informed consent required from patients or representative’s office visit				Establish policy and procedure (P&P) by physicians and hospitals on use of telemedicine that include patient education materials that explain what the patient can expect using telemedicine.	Community-wide education for patients and providers on PHI and maintaining privacy and confidentiality when using telemedicine
(Nieves et al., 2009) *Military Medicine* Pilot Study			All transmission protocols were compliant with HIPAA. It included Tandberg 880 MXP video conferencing equipment and used Integrated Services Digital Network (ISDN) or the hospitals Internet Protocol (IP) network lines with a bandwidth speed of >384 Kilobits per second (Kbps). Audio and image quality were also suitable for use in clinical services.			
(Paing et al., 2009) *Current Psychiatry Reports* Summary of studies/ policy	Families should sign a release for communication and consent for treatment for children; mental health professional should discuss HIPAA provisions with each client as part of the informed consent process. Adolescents were concerned about whether someone could tap into the lines to hear them.	Videotapes of telehealth sessions are an official part of the medical record.	Transmission protocol meets HIPAA requirements	Videotapes kept in secure storage	Practice the 4 C’s: Contracting, Competence, Confidentiality and Control (Koocher) when managing potential risk; Policies must specify who can be included in the session especially those off camera.	More studies on confidentiality, technology issues needed Provide sufficient manpower for technological support
(Peterson & Watzlaf, 2014) *International Journal of Telerehabilitation* Policy	Checklist includes accessibility, amendment, retention of PHI.	Checklist includes requests for PHI, sharing of PHI with other countries and websites.	Checklist includes encryption, user procedures, audit system activity such as username, password (PW), additional authentication, overall assessment	Check cloud based solutions to make sure secure	Checklist includes the need for BAAs for telerehabilitation store and forward companies and if direct identifiers of PHI included a Data Use Agreement (DUA) required under HIPAA	Use Privacy and Security (P&S) checklist to evaluate system/employees before use
(Putrino, 2014) *Current Opinion of Neurology* Opinion			When designing telemedicine systems confidentiality and security are major concern. Designing video game driven telerehabilitation (VGDT) is no exception. Patient data should be de-identified and never stored on the patient’s local device. Data should always be encrypted when streaming across a network.			
(Watzlaf et al., 2010) *International Journal of Telerehabilitation* Policy	Checklist includes accessibility, amendment, retention of PHI, BAAs; Informed consent needed to be signed by patients to include privacy and security issues of telehealth system	Checklist includes requests for PHI, sharing of PHI with other countries and websites, Incident response needed	Checklist includes encryption, user procedures, audit system activity such as username, PW, additional authentication, overall assessment; follow security standards recommended by NIST such as not using username and PW for anything other than telerehabilitation communication, changing it often, using strong usernames and PWs, no computer viruses, and consistently authenticate user communication	Maintain secure transmissions while the session is conducted and when stored and released to internal and external entities		Form team of health and legal professionals to evaluate system for HIPAA, state, and local requirements; educate and train all personnel
(Watzlaf, Moeini, Matusow, et al., 2011) *International Journal of Telerehabilitation* Policy	Examined accessibility and retention of PHI by employees and others.	Examined requests from legal, sharing of information with other countries, websites.	Encryption, antivirus and audit and security system evaluation was examined. 128-bit Advanced Encryption Standard (AES) Secure Real-Time Transport Protocol (SRTP) recommended by NIST. Only 50% of companies reviewed use some form of encryption. 70% of companies made no mention of security evaluation.		Sharing of information was not always addressed in vendor policies	Used a HIPAA compliant checklist for top 10 Voice over Internet Protocol (VoIP) companies Most of the companies did not include all items on the checklist in their policies.
(Watzlaf & Ondich, 2012) *International Journal of Telerehabilitation* Usability Study		Consent for disclosure is needed.	More secure entrance into telehealth system than username and PW.	Impersonation of the system should be prevented. Ensure HIPAA compliance and obtain a BAA between telehealth system and covered entity.	Develop clear, understandable privacy and security (P&S) policies into the patient consent form Policies should describe the P&S of the conferencing session and describe how it will restrict: employee access to technical problems; information to other users; retention of session; and sharing of information with other websites, countries and other third parties without patient consent.	Form a team and use the HIPAA checklist to ensure compliance before using a telehealth system. Review the P&S policies of each system before use. Ask questions of the telehealth company not addressed in the policy.
